# En Bloc Resection and Vascular Reconstruction for Cecal Cancer Involving the Right Common Iliac Artery and Inferior Vena Cava in a Super-Elderly Patient: A Case Report

**DOI:** 10.70352/scrj.cr.25-0478

**Published:** 2026-02-28

**Authors:** Takumi Kato, Takahisa Hirokawa, Ryosuke Niwamoto, Mitsuki Nakazawa, Daiki Sakurai, Kohei Kitamura, Takayuki Saito, Hirotaka Miyai, Masahiro Kimura

**Affiliations:** 1 Division of Gastroenterological Surgery, Kariya Toyota General Hospital, Kariya, Aichi, Japan; 2 Division of Cardiovascular Surgery, Kariya Toyota General Hospital, Kariya, Aichi, Japan

**Keywords:** cecal cancer, en bloc resection, iliac artery, inferior vena cava, vascular reconstruction, femoro-femoral bypass

## Abstract

**INTRODUCTION:**

Complete resection with negative margins (R0) is associated with favorable outcomes in patients with locally advanced colorectal cancer. When tumors invade major vessels, such as the iliac artery or inferior vena cava, curative surgery requires vascular resection and reconstruction, which can be technically demanding.

**CASE PRESENTATION:**

An 88-year-old female presented with abdominal pain and was diagnosed with cecal cancer with para-aortic lymph node metastasis invading the right common iliac artery and inferior vena cava. After laparotomy confirmed resectability, femoro-femoral bypass was performed to preserve lower limb perfusion. This was followed by ileocecal resection with an en bloc resection of the right common iliac artery and partial removal of the inferior vena cava, right ureter, and ovary. Histopathological examination revealed a moderately differentiated adenocarcinoma staged as pT4b (ureter), N2a, and M1a (para-aortic lymph node), corresponding to stage IVa, with negative surgical margins. Her early postoperative course was uneventful, except for a central venous catheter-related infection and transient lower limb edema, both of which were managed conservatively. The patient was discharged on POD 35.

**CONCLUSIONS:**

This case highlights that en bloc resection and vascular reconstruction for colorectal cancer involving major vessels—specifically the right common iliac artery and inferior vena cava—may be performed safely even in super-elderly patients, provided that careful preoperative planning and strict surgical field separation are implemented. Our experience suggests that advanced age alone need not automatically preclude curative-intent surgery when functional status is preserved and the patient strongly wishes to undergo treatment.

## Abbreviation

R0
: complete resection with negative margins

## INTRODUCTION

R0 is a key determinant of favorable long-term outcomes in patients with locally advanced or recurrent colorectal cancer.^1–3)^ When such tumors invade major vessels such as the aorta or iliac arteries, en bloc vascular resection and reconstruction become necessary. However, these combined procedures carry a high risk of complications and perioperative mortality, often rendering the disease unresectable.

Although several reports have demonstrated the feasibility of such combined procedures, especially for recurrent disease in relatively younger patients, evidence remains scarce regarding their application to primary tumors with vascular invasion, particularly in super-elderly patients.

We hypothesized that, with meticulous surgical field separation and procedural adjustments, vascular reconstruction using a prosthetic graft could be safely performed in a potentially contaminated field, simultaneously with bowel resection and anastomosis. Herein, we present a rare case of primary cecal cancer with invasion of the right common iliac artery and inferior vena cava, successfully treated with curative-intent surgery in an 88-year-old patient.

## CASE PRESENTATION

**Fig. 1:**
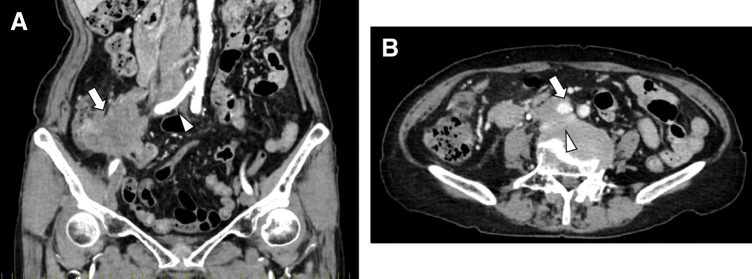
(**A**) Coronal contrast-enhanced CT image on initial presentation showing an exophytic tumor arising from the cecum (arrow), with enlarged para-aortic lymph nodes and invasion of the right common iliac artery (arrowhead). (**B**) Axial contrast-enhanced CT image on initial presentation showing a metastatic para-aortic lymph node invading the right common iliac artery (arrow) and extending anteriorly to involve the inferior vena cava (arrowhead).

**Fig. 2:**
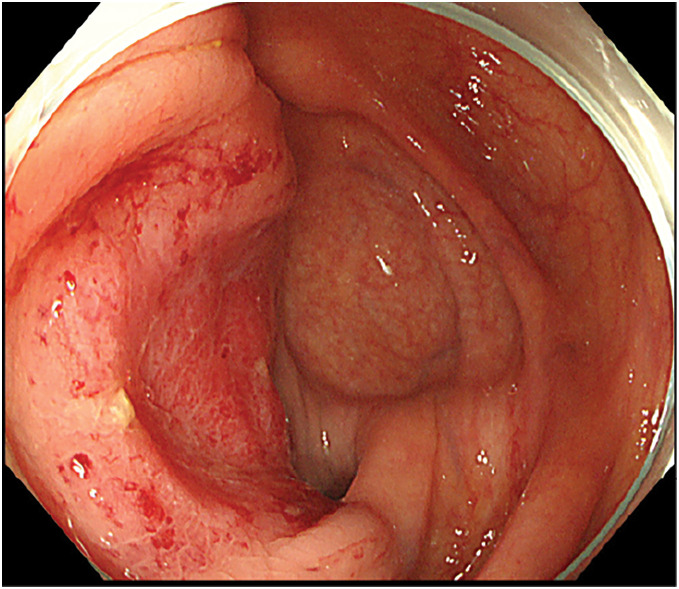
Colonoscopic findings showing a type 2 tumor in the cecum, partially involving the ileocecal valve, without evidence of luminal obstruction.

An 88-year-old female presented to the emergency department of a referring hospital with abdominal pain. CT revealed a cecal mass with invasion of the right ureter, para-aortic lymphadenopathy, and suspected involvement of the right common iliac artery and inferior vena cava (**Fig. 1**). Colonoscopy demonstrated a cecal tumor partially extending to the ileocecal valve (**Fig. 2**), and biopsy showed moderately differentiated adenocarcinoma that was RAS/BRAF wild-type and microsatellite stable. Fluorodeoxyglucose PET-CT revealed no evidence of other distant metastases.

Despite her advanced age, she was fully independent in ADL and had a performance status of 0 on the Eastern Cooperative Oncology Group scale. Her medical history included hepatitis C previously treated with interferon and chronic low back pain, and she was taking ferrous citrate and acetaminophen. Nutritional parameters were acceptable (serum albumin 4.1 g/dL; BMI 18 kg/m^2^). Preoperative cardiopulmonary assessment—including electrocardiography, transthoracic echocardiography, and pulmonary function testing—showed no clinically significant abnormalities. She was assessed as American Society of Anesthesiologists physical status class II and had a Clinical Frailty Scale score of 3.

She was diagnosed with cecal cancer with para-aortic lymph node metastasis and major vascular invasion. Curative resection was considered unfeasible at the initial hospital, and she was referred to our institution for a second opinion. Although para-aortic lymph node metastasis was present, no other distant metastases were detected on imaging, and the disease was considered potentially controllable by complete macroscopic resection. Given that the patient was super-elderly and unlikely to tolerate intensive systemic chemotherapy, we favored curative-intent surgery because an R0 resection appeared achievable. After reevaluation, we considered curative-intent surgery feasible using a femoro-femoral bypass followed by en bloc resection of the involved vasculature and ileocecal resection. The patient provided informed consent, and surgery was scheduled.

### Surgical findings

The procedure began with a midline laparotomy. Given the need for simultaneous tumor resection and vascular reconstruction, strict separation of surgical fields was essential to minimize the risk of prosthetic graft infection in a potentially contaminated operative environment. To facilitate a femoro-femoral bypass via bilateral inguinal incisions, a midline skin incision was made from the upper border of the umbilicus to the level of the anterior superior iliac spine. No intraoperative peritoneal dissemination was observed.

The cecal tumor involved an approximately 5 cm segment of the right ureter, requiring en bloc resection with the right ovary. Ureteroureterostomy was therefore deemed unfeasible. Urinary diversion procedures such as percutaneous nephrostomy or cutaneous ureterostomy, as well as prophylactic nephrectomy, were considered; however, given the patient’s advanced age and the anticipated impact on her ADL, we accepted the risk of unilateral renal function decline and elected to ligate the right ureter without reconstruction. The para-aortic lymph nodes were firmly adherent to the right common iliac artery and the inferior vena cava. Tumor involvement encompassed the right common iliac artery, while sparing both the aortic bifurcation and the bifurcation of the right internal and external iliac arteries. In addition, the tumor had infiltrated the anterior wall of the inferior vena cava at the level of the confluence of the common iliac veins. Because vascular division was deemed technically feasible, en bloc vascular resection was planned to achieve an R0 resection.

A femoro-femoral bypass was performed via bilateral inguinal incisions after temporary closure and redraping of the abdominal incision. A Propaten 8-mm prosthetic graft (W. L. Gore & Associates, Newark, DE, USA) with a removable external ring was anastomosed from the left to the right femoral artery. The groin wounds were completely closed before returning to the intra-abdominal procedure.

**Fig. 3:**
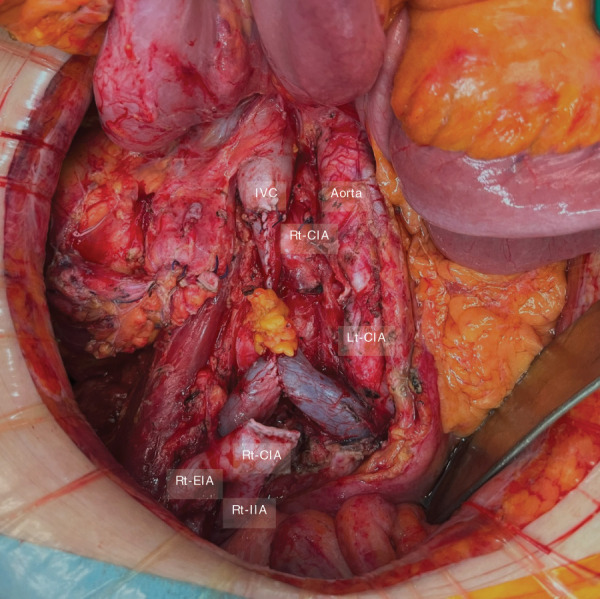
Intraoperative findings after tumor resection. The Rt-CIA was transected proximally at its origin using a surgical stapler, while preserving the bifurcation into the IIA and EIA distally. A portion of the anterior wall of the IVC was resected and closed. The ureter was transected without subsequent reconstruction. EIA, external iliac artery; IIA, internal iliac artery; IVC, inferior vena cava; Lt, left; Rt-CIA, right common iliac artery

The ileocolic vessels were ligated at their origins, and the mesentery was dissected accordingly. The ileum and ascending colon were transected using a linear stapler. The right common iliac artery was divided proximally and at the bifurcation of the right internal and external iliac arteries using a vascular stapler. The tumor-infiltrated segment of the inferior vena cava was divided using Metzenbaum scissors and resected en bloc with the tumor (**Fig. 3**).

**Fig. 4:**
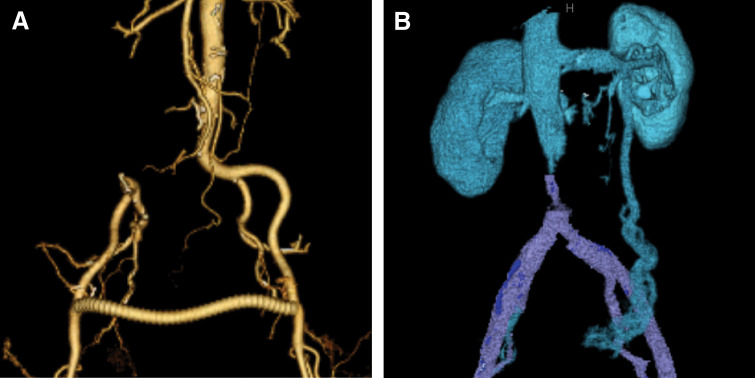
Postoperative CTA showing reconstructed vascular anatomy. (**A**) Arterial reconstruction image demonstrating preserved blood flow to the right internal and external iliac arteries via a femoral-to-femoral bypass graft. (**B**) Venous reconstruction image showing inferior vena cava stenosis, with the left ovarian vein serving as a collateral pathway.

The resultant venous wall defect caused narrowing of the inferior vena cava lumen. Because the defect was amenable to primary closure and considering the risk of prosthetic infection in a potentially contaminated field, venous reconstruction was not performed; instead, the inferior vena cava was closed primarily (**Fig. 4**). Total operative time was 6 h 49 min, with an estimated blood loss of 570 mL.

### Histopathological findings

Histopathological examination revealed that the cecal tumor exhibited a diffuse infiltrative growth pattern, leading to the loss of normal bowel wall architecture. The tumor was predominantly a moderately differentiated tubular adenocarcinoma with evidence of direct invasion into the adjacent right ureter. Based on the final pathological evaluation, the tumor was classified as type 4 and demonstrated serosal and ureteral invasion corresponding to the depth of invasion of pT4b. Lymphatic and venous invasion were observed. Both the proximal and distal surgical margins were free of tumor cells. Regional lymph node metastasis (pN2a) was observed, and a para-aortic lymph node (station #216) was positive for metastatic disease, indicating distant lymphatic spread (pM1a). Collectively, these findings correspond to a final pathological stage IVa.

### Postoperative course

The postoperative course was generally favorable. The patient developed a central venous catheter–related bloodstream infection, which resolved promptly after catheter removal and antibiotic therapy.

On POD 18, the ankle–brachial index was 1.09 on the right and 1.13 on the left, indicating preserved lower limb perfusion. Lower extremity edema developed postoperatively and was attributed to inferior vena caval stenosis. The edema peaked on POD 22 and gradually improved with conservative management. The patient was discharged in stable condition on POD 35.

At 3 months postoperatively, no recurrence was observed; the lower extremity edema had resolved, and the patient had returned to her usual daily activities. Her abdominal pain resolved, oral intake was adequate, and she expressed satisfaction with the postoperative QOL. At 6 months after surgery, contrast-enhanced CT and fluorodeoxyglucose PET-CT revealed recurrent disease involving the right upper lobe of the lung, mediastinal lymph nodes, para-aortic lymph nodes, and the right external iliac lymph node. After discussion with the patient, systemic chemotherapy was not pursued, and best supportive care was selected.

## DISCUSSION

Achieving R0 resection in cases of locally advanced or recurrent colorectal cancer is closely associated with improved long-term survival outcomes.^1–3)^ However, when tumors invade major vascular structures, such as the aorta or iliac arteries, curative resection requires both en bloc tumor removal and complex vascular reconstruction, which is traditionally associated with high rates of postoperative complications and limited survival benefit.^4)^

Recent reports suggest that, in selected cases where surgery remains the only potentially curative option, combined oncologic resection and vascular reconstruction can be performed safely and effectively.^4–8)^ For example, Abdelsattar et al. reviewed 12 cases of locally advanced or recurrent colorectal cancer involving vascular invasion of the aorta or iliac arteries. Although postoperative complications occurred in 9 cases, there were no deaths within 30 days.^5)^ These findings support the feasibility and potential benefit of R0 resection even in the presence of vascular involvement; however, it should be noted that all patients in that series had recurrent disease.

In contrast, reports of primary colorectal cancer requiring major vascular resection and reconstruction remain extremely rare, with only 2 published cases to date.^6,7)^ While our surgical strategy closely resembles those prior reports—utilizing extra-abdominal bypass, strict field separation, and 1-stage resection—our case adds incremental novelty by demonstrating feasibility in a super-elderly (88-year-old) patient with inferior vena cava involvement. The clinical characteristics and surgical details of these cases are summarized in **Table 1**.

**Table 1:** Summary of reported cases of colorectal cancer with major arterial invasion treated with vascular reconstruction and en bloc resection

Author (year)	Age/sex	Primary site	Artery involved	Bypass type	Postoperative complications	Chemotherapy (Pre/Post)	Outcome
Kubota et al. (2021)^6)^	29/Male	Cecum	Right external iliac artery	Femoro-femoral bypass	None	Yes/Yes	No recurrence (2 years)
Enari et al. (2025)^7)^	73/Male	Sigmoid colon	Left common and external iliac arteries	Axillo-femoral bypass	None	Yes/No	No recurrence (10 months)
Present case	88/Female	Cecum	Right common iliac artery	Femoro-femoral bypass	None	No/No	Recurrence (6 months)

Among these 2 reported cases, Kubota et al. described a 29-year-old male with cecal cancer invading the right external iliac artery.^6)^ A femoro-femoral bypass was performed, followed by 1-stage resection and bowel anastomosis. Enari et al. reported the case of a 73-year-old man with sigmoid colon cancer infiltrating the left common iliac artery.^7)^ In that case, an axillo-femoral bypass was performed, and resection with anastomosis was likewise completed in a single operation. In both cases, vascular reconstruction was performed to preserve lower limb circulation, and techniques were employed to avoid exposing the prosthetic graft within the abdominal cavity. No postoperative complications were reported. In addition, chemotherapy was administered either preoperatively or postoperatively in these 2 cases.

Most previously reported cases of primary or recurrent colorectal cancer with vascular invasion involved relatively young patients. In contrast, our case represents a rare example of curative-intent surgery performed in a super-elderly patient aged 88 years. Despite her advanced age, the patient’s ADL were preserved and she strongly wished to undergo surgery; therefore, curative-intent resection was pursued.

The optimal management of para-aortic lymph node metastasis from colorectal cancer remains controversial. Nevertheless, for synchronous para-aortic lymph node metastasis without other distant metastases, an R0 resection strategy has been reported to yield acceptable outcomes in selected patients.^9)^ Moreover, recent retrospective data suggest that surgical intervention for resectable para-aortic lymph node metastasis may be associated with improved overall survival compared with nonsurgical management, although these findings should be interpreted cautiously given the retrospective design and potential selection bias.^10)^ Accordingly, indications should be individualized and determined through thorough multidisciplinary discussion, balancing oncologic benefit against treatment burden, particularly in super-elderly patients.

If surgery had not been performed, alternative options would have been systemic chemotherapy or observation. Given the patient’s age, chemotherapy might have been suboptimal due to limited tolerability and the risk of adverse effects that could compromise QOL. Observation alone would have likely led to symptomatic progression and further deterioration in QOL. Thus, surgical resection was considered the most appropriate option for both oncologic control and preservation of QOL. Postoperatively, she became pain-free and maintained adequate oral intake, indicating that resection may have provided meaningful patient-centered benefit in addition to oncologic intent.

No postoperative adjuvant chemotherapy was administered. After shared decision-making with the patient, adjuvant chemotherapy was omitted because of concerns regarding tolerability and potential deterioration in QOL. According to a retrospective study by Nozawa et al., adjuvant chemotherapy following resection of metastatic para-aortic lymph nodes is associated with improved progression-free survival and overall survival, suggesting a potential prognostic benefit.^11)^ However, other studies have shown that, even in elderly patients who underwent resection, adjuvant chemotherapy did not lead to a significant survival improvement.^12)^ Given the patient’s age and overall vulnerability, we decided not to administer adjuvant chemotherapy.

A major concern in simultaneous gastrointestinal and vascular surgery is prosthetic graft infection, which can be life-threatening. Therefore, strict separation of the clean (vascular) and contaminated (gastrointestinal) fields is essential.^13)^ In our case, a femoro-femoral bypass was chosen to avoid intra-abdominal graft placement and to maintain complete field separation. Preoperative planning, including incision design and stepwise sequencing, was implemented to minimize the risk of contamination.

Furthermore, mesenteric vessel ligation was deferred until vascular reconstruction was complete to reduce the risk of graft infection and ensure adequate bowel perfusion. Once the vascular field was closed, bowel resection and anastomosis were performed as a separate step. These precautions likely contributed to the absence of graft-related complications and the favorable postoperative course observed in our patient.

A limitation of this report is the relatively short follow-up period, and longer observation is necessary to evaluate long-term oncologic outcomes.

## CONCLUSIONS

Simultaneous vascular reconstruction and oncologic resection may be safely performed in carefully selected patients with colorectal cancer and major vascular invasion. Although similar combined procedures have previously been reported mainly in younger patients or in the setting of recurrent disease, our case suggests that this strategy can also be feasible in super-elderly patients when functional status is preserved and meticulous perioperative planning is undertaken. In otherwise unresectable cases, this approach may offer curative potential and can be considered as one of the therapeutic options following thorough multidisciplinary discussion.
